# A Qualitative Study to Examine Feasibility and Design of an Online Social Networking Intervention to Increase Physical Activity in Teenage Girls

**DOI:** 10.1371/journal.pone.0150817

**Published:** 2016-03-02

**Authors:** Gisela Van Kessel, Madeleine Kavanagh, Carol Maher

**Affiliations:** University of South Australia, School of Health Sciences, Adelaide, Australia; University of Bath, UNITED KINGDOM

## Abstract

**Background:**

Online social networks present wide-reaching and flexible platforms through which to deliver health interventions to targeted populations. This study used a social marketing approach to explore teenage girls’ perceptions of physical activity and the potential use of online social networks to receive a physical activity intervention.

**Methods:**

Six focus groups were conducted with 19 Australian teenage girls (ages 13 to 18 years) with varying levels of physical activity and socioeconomic status. A semi-structured format was used, with groups discussion transcribed verbatim. Content analysis identified emergent themes, with triangulation and memos used to ensure accuracy.

**Results:**

Physical activity was most appealing when it emphasised sport, exercise and fitness, along with opportunities for socialisation with friends and self-improvement. Participants were receptive to delivery of a physical activity intervention via online social networks, with Facebook the most widely reported site. Participants commonly accessed online social networks via mobile devices and particularly smartphones. Undesirable features included promotion of physical activity in terms of walking; use of cartoon imagery; use of humour; and promotion of the intervention via schools, each of which were considered “uncool”. Participants noted that their parents were likely to be supportive of them using an online social networking physical activity intervention, particularly if not promoted as a weight loss intervention.

**Conclusion:**

This study identified key features likely to increase the feasibility and retention of an online social networking physical activity intervention for teenage girls. Guidelines for the design of interventions for teenage girls are provided for future applications.

## Background

Physical activity has been associated with many health and social benefits for children, including healthy body weight, and greater bone density, fitness and self-esteem [1]. Despite these benefits, a recent Australian Bureau of Statistics survey found that just 19% of Australian children aged 5–17 years met the physical activity guidelines of 60 minutes of moderate-to-vigorous physical activity (MVPA) per day [2].

Children’s physical activity patterns are recognised to vary on the basis of key demographic factors, such as age and sex [2, 3]. Teenage girls have been identified as a group particularly at risk for physical inactivity, getting less physical activity than boys the same age, and younger girls [4], which is thought to be due to a wide range of social, cultural and biological factors [5, 6].

Physical activity interventions targeting teenagers have typically been delivered via schools [7]. Schools offer a convenient means of reaching a large number of children from a range of socio-demographic backgrounds [8]. However, anecdotally, delivery at schools also faces key barriers, such as overburdening of teachers, overcrowding of curricula and difficulty targeting specific segments of the school population (e.g. only girls).

The enormous popularity of online social networks offers a new medium through which to reach targeted audiences, such as teenage girls. Possible advantages of delivering interventions via online social networks include ready accessibility, the potential for social support, and excellent potential for scalability and mass-delivery [9]. Two recent systematic reviews on use of online social networks for health behaviour change identified three studies [10–12] targeting US college students, but no studies targeting younger populations.

Social marketing has been used to develop relevant and appealing mass health messages. A social marketing approach guides intervention design to consider elements such as product, price, promotion and placement. Social marketing seeks to influence behaviour by providing something of value as a trade for acting on a particular message [13]. In order to successfully promote health messages, intervention designers need to understand what is valued, what can be traded and what it competes with, from the intended users’ perspective.

To develop this understanding of perspectives, social marketers use segmentation to divide the population of interest into subgroups based on shared characteristics, such as demographics, psychographics and behaviour [13]. To date, research addressing physical activity with a social marketing approach has predominantly focussed on adult populations [14–20]. Subitha et al. [14] used a social marketing approach to design a community-based physical activity intervention for rural Indian adults, which achieved high program uptake and attendance. Similarly, Withall and colleagues’ social market campaign in a disadvantaged US community led to increased exercise session attendance compared to pre-campaign attendance and a control community [17].

To date, less work has been done using social marketing to increase physical activity in younger populations, but there are indications that tweens (9–13 year olds) have different perceptions and values from adults [21], highlighting the importance of undertaking preparatory work with the intended population. For example, while adults value “exercise”, tweens value enjoyment and spending time with friends [21] and being fit and appealing to the opposite gender [22]. It is also important to note that different subgroups within a population of teenage girls may have different perceptions regarding benefits and costs which should be taken into account in order to maximise effectiveness of future physical activity interventions.

This study aimed to address these needs using a social marketing approach to explore teenage girls’ perceptions of physical activity, with a view to informing the design and marketing of an online social networking physical activity intervention for teenage girls. We aimed to identify perceived benefits and barriers to physical activity as well as key design elements (online social network sites, structure, features, and styling) for the development of an online social networking physical activity intervention.

## Methods

### Study design

A qualitative focus group design was selected as the most suitable method for capturing participants’ perceptions regarding an online social network-delivered physical activity intervention [20]. The study used a social marketing approach informed by Exchange Theory, which suggests that in order for teenage girls to increase their physical activity, there must be a transfer or trade of something of value [13]. The perceived benefits (e.g. fitness) offered to teenage girls in exchange for increasing physical activity must be greater than any perceived costs (e.g. loss of social time). Consequently, we needed to understand not only what the perceived benefits and costs were but which costs were too high and which benefits were too low [13].

Ethical approval was provided by the University of South Australia Human Research Ethics Committee, and teenage participants and their parents provided written informed consent prior to participation.

### Participants & recruitment

We defined teenage girls as those aged between 13–18 years, residing in Adelaide, Australia, and fluent in English. Recruitment occurred through professional and personal contacts of the research team. Purposive sampling was employed to recruit participants with varied sociodemographic characteristics and personal interests. We were interested to explore perspectives of girls who were younger teenagers or older teenagers; Caucasian, or Aboriginal; low or high socioeconomic area, as well as girls who were or were not interested in sport. We sought groups of girls who were acquainted with each other prior to the focus group to allow maximal discussion and sharing of opinions.

### Data collection

All six focus groups were conducted by the same two researchers (MK and CM). Focus groups two, three and four were conducted at the University of South Australia, focus group one at a sport stadium, focus group five at the home of a participant and focus group six in a public library meeting room. A question guide was used to form the semi-structured focus groups, lasting for approximately one hour. There were three general sections to each focus group, (1) general views on physical activity, (2) views on the concept of an online social networking intervention, and (3) suggestions for the specific content and design of a potential online social network intervention (S1 Text. Focus group schedule). Note that Social Marketing Theory constructs were not explicitly used to develop the question guide. Rather, they were used to guide data analysis. The focus group began with a psychometric exercise that asked the girls to stand at a distance from a mark on the floor to indicate their response to two questions regarding their beliefs about physical activity. Following this, participants sat around a table, and a series of questions explored their views regarding physical activity facilitators and barriers, along with their use of the internet and online social networks. Next, an existing online social networking intervention designed for adults [23] was demonstrated to the girls on an iPad, and they asked to comment on its features and whether they were likely to appeal to teen girls. Finally, the girls were shown some mocked up graphic designs for an online intervention targeting teen girls (S1 Fig. Test social media material—design), along with mocked up written content in three distinct styles (enthusiastic vs educational vs inspirational) and a list of mocked up daily tips for physical activity. (S2 Text. Test social media material—tips). Participants were asked to rank the various alternate graphic designs, and daily tips, and provide feedback on the linguistic style of the written content.

### Data coding and analyses

A manual, inductive and iterative content analysis of the transcripts was undertaken. This involved the three researchers independently and repeatedly reading and assigning codes at the sentence level, by selecting a word that captured the content of the sentence. The researchers reviewed each others’ coding and discussed varying views or disagreements to reach consensus. One researcher (GVK) collated the codes into categories such as product, price, promotion and placement developed from Social Marketing Theory to organise the code list. This level of the analysis was reviewed by the remaining two researchers and no further changes were deemed to be required.

Rigour was supported by (1) writing of memos on each focus group to capture overall impressions, as well as (2) triangulation (i.e. the three researchers undertook coding independently, and then came together to discuss emerging codes, categories and themes until consensus was reached) and (3) actively seeking negative cases and contradictory data.

## Results

A total of 19 teenage girls were recruited into six focus groups. Focus groups were purposefully recruited to capture diversity within the teen girl population, as summarised in Table 1.

**Table 1:** Participant Description.

Focus group number	Key population representation	No. of participants	Average age	Race	SES*
1	Metropolitan members of a sports club	4	15.5	Caucasian	6-Moderate
2	Inner metropolitan Private school	3	17	Caucasian	9-High
3	Metropolitan Public school	3	14	Caucasian	8-High
4	Outer metropolitan area	4	14	Caucasian	5-Moderate
5	Metropolitan Public school	3	13	Caucasian	1-Low
6	Boarders from remote area boarding to attend public school.	2	14	Aboriginal	Unknown^$^

• *SES determined by SEIFA average rank within Australia deciles as calculated by post code [24]

• ^$^Boarding post code not indicative of SES

Detailed findings are discussed below, while data is presented in tables with the themes ranked to indicate spread across focus groups (S1 Table. Qualitative analysis codes).

### Product

Within this study, “product” was considered to be the package of benefits that arise from undertaking physical activity, including augmented products such as social engagement with friends [25]. Physical activity was identified by participants primarily as fitness and organised sport (e.g. running, team sports). Many participants identified that they did not consider walking or day-to-day activities (e.g. housework) as forms of physical activity. The reported physical benefits of undertaking physical activity included improved fitness, attractiveness, and to a limited extent, health. Health benefits seemed to have been mentioned by participants as an obligatory advantage of exercising, rather than a primary motivator. In addition, participants seemed reluctant to raise attractiveness as a motivator, as it was not seen as a socially acceptable motivator (Table 2, quote 1).

**Table 2:** Table of quotes.

Reference	
**Product**	
**Quote 1**	“It depends, like, aspire to, kind of [be] fit I think, obviously not like a perfect girl, cause I know when people see like a lot of models [agreement by others in the group], it’s kind of like aspire to be that [model] but it’s kind of bad in a way” FG1-1
**Quote 2**	“because it helps you like in life, like later in life, and also it makes you feel good later in life as well because if you’re a winner or something like that, it makes you feel good inside” FG5-16
**Quote 3**	“It’s like when you play games of netball against your friends you want to win more than you usually do compared to someone you don’t know… So you’re probably more likely to try harder and be more competitive against your friends because you want to win” FG1-3
**Quote 4**	“I enjoy the social side of things and I prefer to do a sport like a team sport over having to run on my own. Like, I find being with other people motivates me” FG1-2
**Price**	
**Quote 5**	“Well I just started a job so they’ve been giving me lots of shifts and now it’s to find time between that and school and being busy” FG1-3
**Quote 6**	“a downside to competition is if you got low steps you might feel bad about yourself. Why can’t I do as many as they did?” FG5-15
**Online social networks as a promotion channel**	
**Quote 7**	“there’s about four girls out of a total of like 120 [in my year level at school] who don’t have Facebook” FG1-4
**Quote 8**	“basically everyone uses it on their phone” FG1-4
**Quote 9**	“it’s not like it’s a weight loss program, that parents could be kind of a bit cautious about” FG1-1
**Placement of product**	
**Quote 10**	“…the school’s saying do it, don’t do it cause it’s not cool” FG5–16
**Features of an online social network based intervention**	
**Quote 11**	“It would be funner with people you know because it’s more competitive” FG1-2
**Quote 12**	“getting teenagers out of their comfort zone… not using social media and network, like getting out there and doing something
	Interviewer: So it would be good to get your friends out and help them do something?
	Yeah” FG6 19
**Quote 13**	“if I was a leader I’d probably feel like I had to get everyone to do it and it was my fault if they didn’t” FG4-9
**Quote 14**	“it would be cool if you got a reward” FG2-6
**Quote 15**	“if I kept forgetting, I’d just be like oh, I’ve forgotten the last 2 weeks, that would make me drop out. And I’d feel bad” FG3-8
**Quote 16**	“like if one of our family members was really sick and they needed us to help them and if one of our family members passed away” FG6-19
**Quote 17**	“if nothing new happened, like if there was no updates or anything I’d probably get bored with it. I’d want it to like change design every now and again”
**Features of an online social network program**	
**Quote 18**	FG2-6 “they’re quick and eye catching and they’re not that detailed…it looks cool” FG5-16
**Quote 19**	“this is for fat people” FG3-8
**Quote 20**	“it feels a bit kiddy kind of thing, the cartoon” FG1-3

Note quotes are identified by focus group number and participant number e.g. FG1-1 is focus group 1 participant 1

Physical activity was reportedly facilitated in this group of teenage girls through intrinsic factors, such as a commitment to goals, a sense of self-efficacy, self-motivation or achievement of personal best. (Table 2, quote 2). In addition, extrinsic factors such as routines, training programs, classes and paying for gym membership, parental direction and in particular, friendly competition with friends, appeared to be important influences (Table 2, quote 3). Physical activity was particularly valued as an avenue to socialise with friends, and as such, was seen to contribute to enjoyment and happiness (Table 2, quote 4).

### Price (perceived costs)

A social marketing approach defines price as the cost perceived in undertaking physical activity [26]. The teenage girls recognised a number of barriers to participating in physical activity. Time for physical activity had to compete with part-time employment, school and homework demands (Table 2, quote 5). Other barriers included injury, bad weather, and lack of transport, money or safety.

Participants reported that partaking in physical activities contributed to positive feelings towards themselves, but conversely, they also worried about not keeping up, or poor performance, leading them to feeling like they may be a burden or inadequate (Table 2, quote 6).

### Online social networks as a promotion channel

Promotion considers which channels (TV, radio, internet etc.) may be effective for communicating a message about physical activity [25]. We were particularly interested in the feasibility of a physical activity intervention for teenage girls delivered via online social networks. Study participants reported that Facebook was, by far, the more common online social networking site used, with many of the girls reporting that they used Facebook many times a day (Table 2, quote 7). Instagram was reported by a minority of participants, however, those who used it, favoured it over Facebook. No participants reported using Twitter. Participants reported accessing online social networking sites predominantly via mobile phones, (Table 2, quote 8).

Participants reported that their parents, and their peers’ parents, had some concerns about cyber-safety e.g. bullying and internet addiction. However, most participants agreed that once informed, their parents would likely be highly supportive of their participation in a physical activity intervention delivered via online social networks (Table 2, quote 9).

### Placement of product

Social marketing suggests that how products are placed or distributed may create opportunities [25]. Participants suggested an online social networking physical activity intervention would have enhanced credibility if it was clear that it had emanated from a university. The potential for dissemination of an online intervention via schools received a mixed response, with some participants feeling that schools would facilitate uptake, but others perceiving programs run by schools as being “not cool” and that this would be a strong deterrent from taking up the program (Table 2, quote 10).

### Features of an online social network based intervention that might facilitate an increase in physical activity

Potential features of an online social networking program were presented to the teenage girls during the focus groups, such as an online team format (comprising either existing friends, or strangers) and use of pedometers to track daily physical activity. The teenage girls confirmed that friendly-competition, goal-setting and group aspects would be appealing to them (Table 2, quote 11). The Australian Aboriginal girls also noted that a team structure would assist people other than themselves to benefit (Table 2, quote 12). Whilst participants clearly liked a team format, when they explicitly asked about their willingness to adopt a leadership role to form a group, they expressed reluctance to do so (Table 2, quote 13).

The participants expressed a desire for high levels of feedback during the program, such as regular feedback on their own performance, notifications on their friends’ achievements and feedback on multiple metrics, such as daily steps and energy (calorie) use. Additionally, extrinsic rewards were appealing (Table 2, quote 14).

In terms of barriers, the most critical factor threatening the success of an online social networking physical activity program was the risk of forgetting to use it and subsequently falling behind (Table 2, quote 15). The Australian Aboriginal girls identified that their commitments to their family may also act as a barrier to program adherence (Table 2, quote 16). Many of the participants recognised that it was difficult to maintain interest in physical activity or in any particular website/app as novelty wears off (Table 2, quote 17). They had a number of suggestions to maintain novelty that included keeping the exercises simple and easy to perform at home, making sure program content varied from week to week, offering information on progressive forms of exercise and using music to make exercise enjoyable.

### Design features of an online social networking program

The focus groups were presented with a variety of graphical themes and colour schemes which had been created by a graphic designer, and asked to comment on them. The girls identified that the design should meet criteria such as using light, bright colours, having a plain background, being easy to read with clear fonts, and using a simple, eye-catching design (Table 2, quote 18). Although participants stated that they preferred imagery of teenage girls that portrayed “realistic” and “not too skinny” body shape, when presented with a range of images, participants preferred those which depicted slender, attractive models. Images of more overweight models were not preferred, (Table 2, quote 19). Illustrated images of teenage girls were perceived as childish and unappealing (Table 2, quote 20).

The participants expressed a desire for the program branding to have a clear “fitness” theme, and did not like designs that were feminine without being of sport/fitness in nature (“too girly”) or were obviously youth-orientated (e.g. one of the design options used graffiti imagery and was criticised for “trying too hard to be cool”). In regards to the written content of the intervention, content which was uplifting, motivational, and educational was preferred, whereas content which was humorous was deemed “uncool”.

## Discussion

This study explored teenage girls’ perceptions regarding physical activity and software design features and content for an intervention to promote physical activity delivered using online social networking with a social marketing approach. We found that teenage girls have different perceptions from adults and tweens about physical activity and participating in groups that could influence the success of any health promotion campaign to this group.

A number of findings contrasted with those identified by previous researchers using a social marketing approach with either younger or older audiences. For example, we found that promotion of physical activity via lifestyle activities such as chores and walking is both unfamiliar and unappealing to teenage girls, whereas this is a popular mechanism for adults [26, 27]. Furthermore, while Wong et al [21], identified fun as a key motivator of physical activity for tweens, our participants appeared to be more serious and orientated towards self-improvement. These differences might be explained by the different life stages. In educational contexts, younger children are recognised to rely heavily on intrinsic motivators such as enjoyment [28], whereas as they age, extrinsic motivators become more important [29]. In addition, adolescence is a period in which individuals consciously consider and shape their self-identity [30], which may reflect their preference for intervention materials with a self-development tone.

This study identified “coolness” as a key factor likely to impact intervention uptake or rejection, which was also identified in Wong and colleagues’ study in tweens [21]. In particular, our participants expressed disdain for potential program features they considered to be “uncool”. These included cartoon images, humorous language, and branding that was perceived as being “try-hard” (e.g. branding featuring graffiti). These preferences may reflect adolescents’ self-concept as emerging adults [30]; cartoon images and playful language were perceived as being incongruent with this.

Similarly, findings revealed that delivery of a program via schools may be off-putting to a large proportion of teenage girls. This contrasts with the current reality of intervention delivery. Two previous reviews of physical activity interventions targeting teenagers reported that programs to date have been predominantly school-based [7, 31]. Interestingly, school-based physical activity programs have typically shown limited transfer to non-school activity time and long term effectiveness [31]. Taken together with the findings of our study, it appears important that avenues for delivering physical activity interventions outside of school be considered.

Findings highlighted the importance and dynamics of peer networks for teenage girls. Consistent with previous literature, findings suggested that teenage girls are heavy users of online social media. Furthermore, the girls expressed strong interest in undertaking the program with their friends, which may assist in disseminating an online social networking-based intervention. However, peer-related issues also raised potential threats to a program’s success, with girls noting that their friends’ use of a program would influence their own use (e.g. if their friends stopped using it). The girls expressed reluctance to assume a leadership role, which is consistent with previous research suggesting that teenage girls tend to assimilate in order to achieve centrality within their peer network, in contrast to teenage boys who are more likely to seek individuation and uniqueness [32].

Strengths of the current study are that it recruited teenage girls who ranged widely in terms of socio-demographic characteristics and physical activity behaviour, and that it was successful in prompting in-depth discussion of the topics of interest. In particular, the study identified a number of factors which are likely to be highly important to the success or otherwise of an online social networking intervention for teenage girls (for example, the invitation structure used to recruit new users, and elements of the app branding and content, and teenage girls’ strong desire for feedback regarding their physical activity performance). To our knowledge, these findings are novel, and add considerably to existing scientific knowledge in this area. Further strengths are that the study was embedded in a strong theoretical framework (Social Marketing Theory and Exchange Theory) and that analyses were undertaken with triangulation, reducing the risk of bias compared with analyses undertaken by one researcher.

This study examined feasibility and design of an online social networking intervention in a group of Australian teenage girls but there is a risk that the study’s findings are not generalizable because all of the focus groups were undertaken within one city of Australia. Values regarding physical activity may vary in elsewhere in the world value, and are likely to be influenced by culture and resources.

This study identified a number of characteristics regarding promotion of physical activity to teenage girls which are likely to be useful to other researchers and practitioners in this field:

1 Physical activity as a product should emphasise sport, fitness and exercise, and opportunities for social interaction with friends.
2 Messaging should emphasise self-improvement and benefits for wellbeing and attractiveness.
3 To maximise appeal, a program’s branding should feature photographic images of attractive, slender, healthy-looking young women.
4 Features which are perceived as “uncool” or “trying to be cool”, are strongly off-putting for this population. Strategies and features to be avoided (or used with caution) include promotion of a program via schools, attempts at use of humour in program content, marketing of physical activity in terms of walking or chores, and use of cartoon images in the intervention’s branding.

Furthermore, key findings, specific to the feasibility and design of an online social networking physical activity intervention for teenage girls were:

5 Confirmation that teenage girls are heavy online social network users, and of Facebook in particular.
6 Such sites are typically accessed using smartphones, thus health interventions using online social networks should be optimised for smartphones.
7 The ability to undertake an intervention with friends is appealing, but mechanism used to achieve this via online social networks should not require a participant to take on a leadership role within their friendship group.

Teen girls are an at-risk population for physical inactivity, and intervention efforts to date have experienced difficulty bringing about behaviour change. Thus, we hope these insights and recommendations will assist in the development of effective, targeted interventions into the future.

## Conclusion

This study found that online social networks appear to offer considerable promise as a feasible and appealing platform for delivering a physical activity intervention to teenage girls. Such intervention programs should emphasise fitness and sport, along with the ancillary social, self-improvement and physical attractiveness benefits of physical activity. Teenage girls are a highly discerning population, and specific elements of graphical style and branding of a program are likely to have a strong attracting/detracting influence on whether or not an online intervention will be used by this population.

## Supporting Information

S1 Fig Test social media material—design. (PDF)

S1 Table Qualitative analysis codes. (PDF)

S1 Text Focus group schedule. (PDF)

S2 Text Test social media material–tips. (PDF)
