# Supplementary figures and images for: A Qualitative Study to Examine Feasibility and Design of an Online Social Networking Intervention to Increase Physical Activity in Teenage Girls
Source: PLoS One. 2016 Mar 2;11(3):e0150817. doi: 10.1371/journal.pone.0150817 (PMC4774869; doi:10.1371/journal.pone.0150817)

1

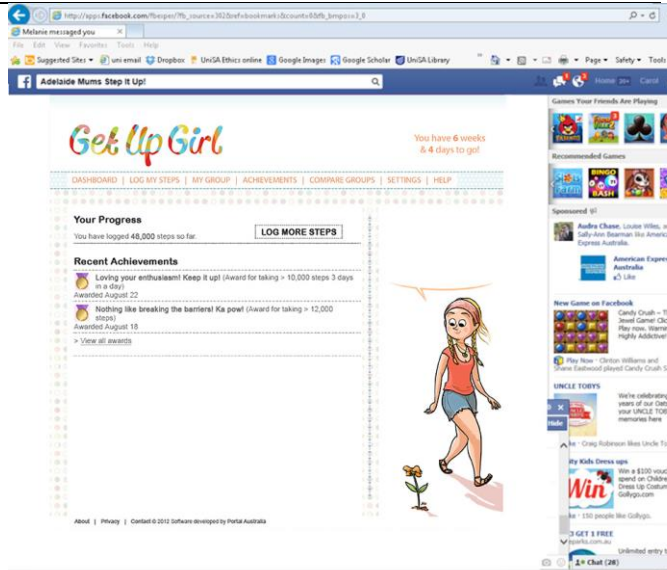

2

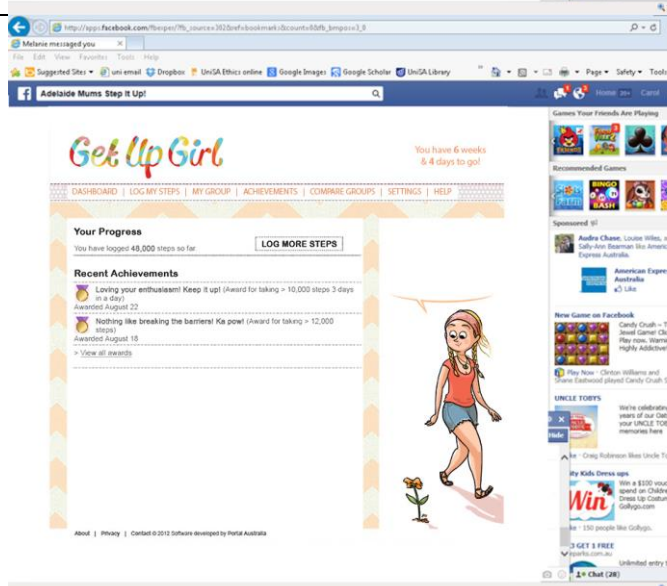

3

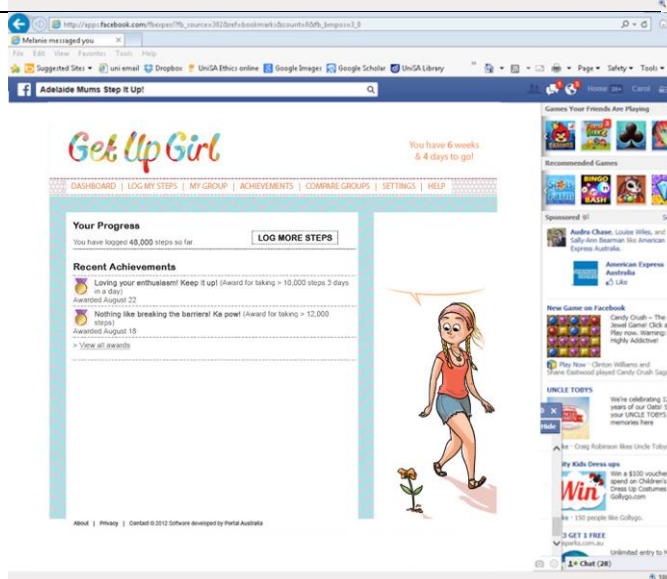

4

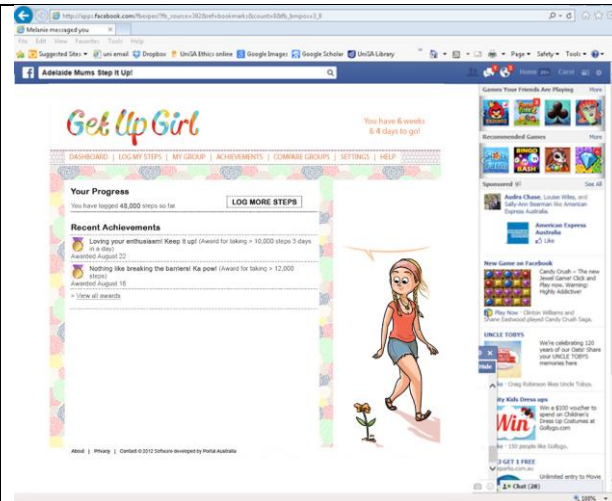

5

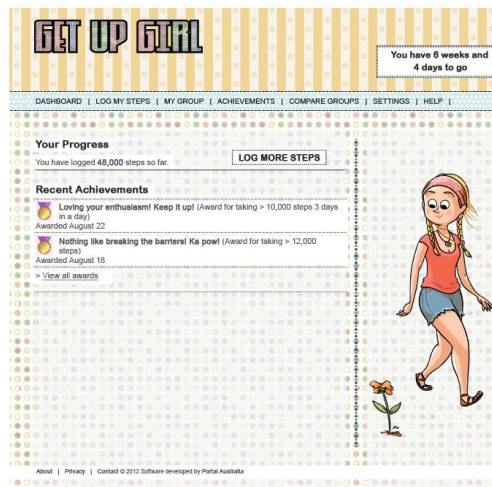

6

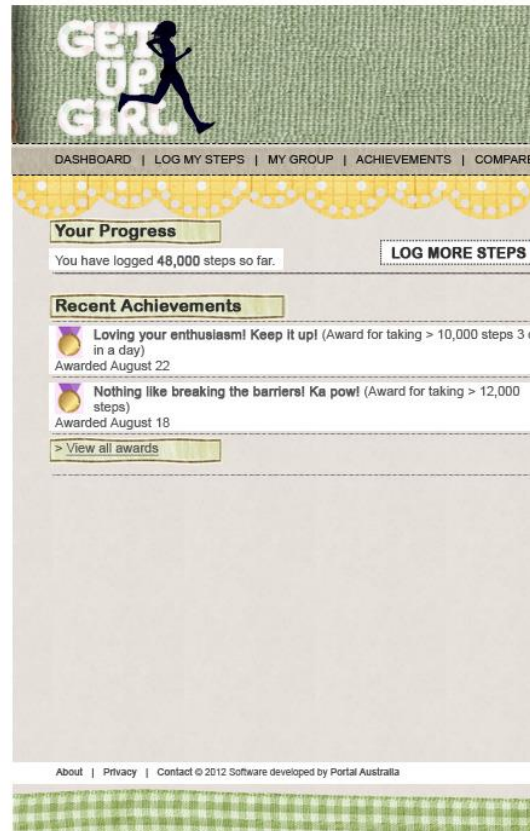

7

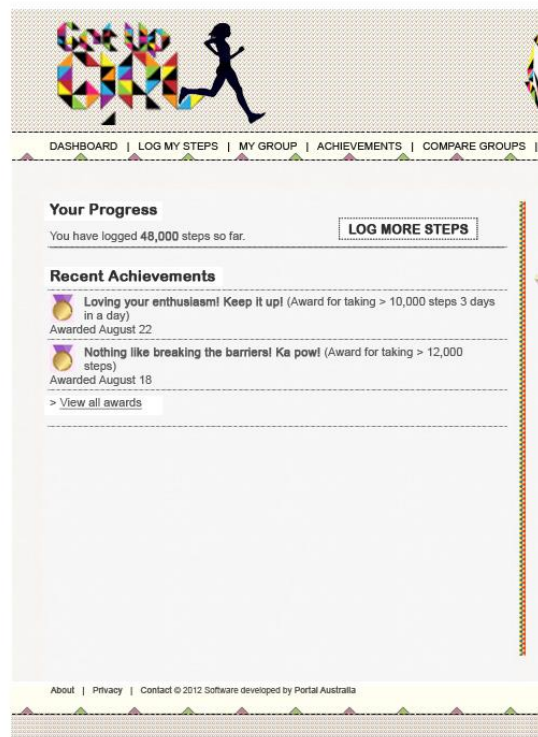

8

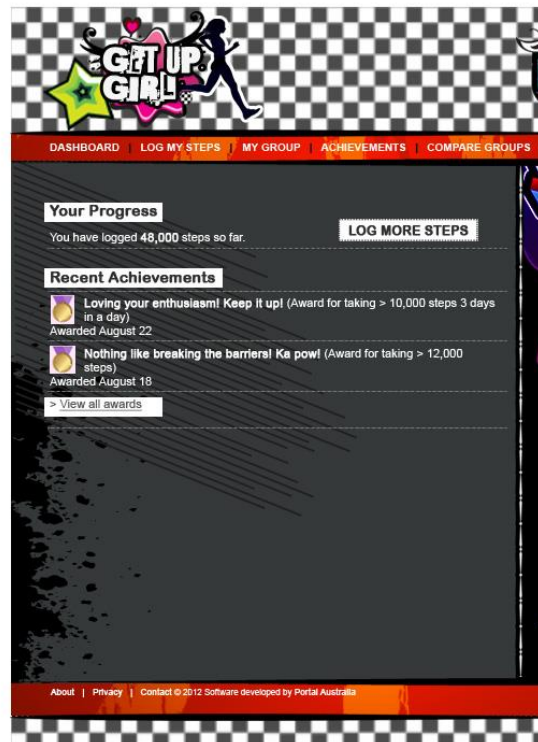

9

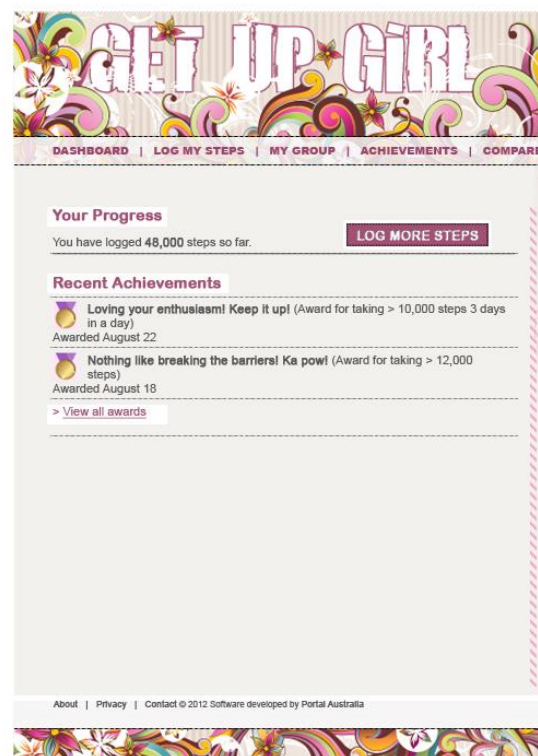

10

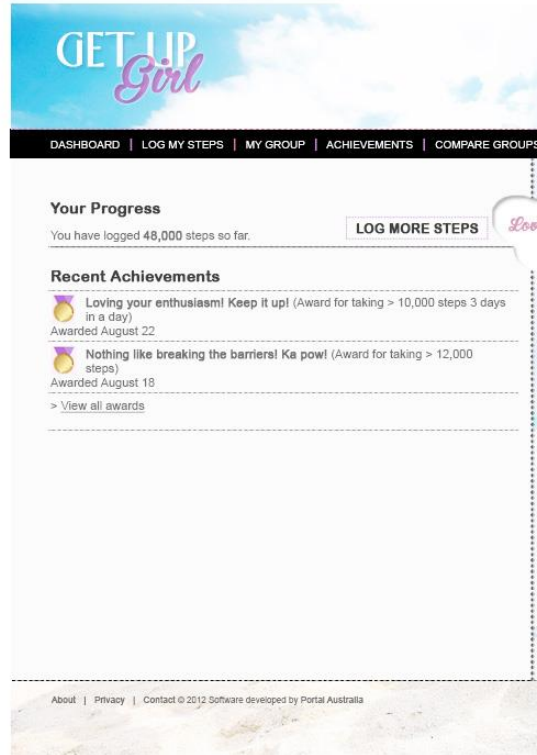

Graphics

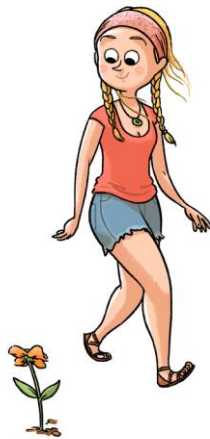

Graphics

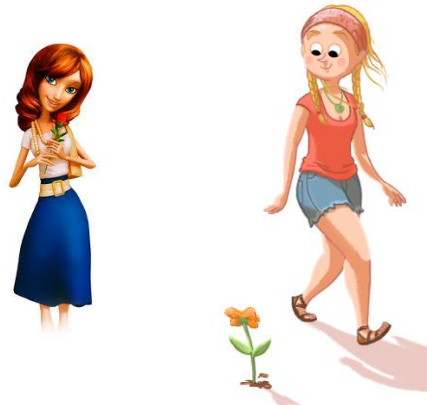

Graphics

Graphics

Get Up  
Girl

Get Up Girl

Supplement: S1 Fig — (PDF) [file pone.0150817.s001.pdf]
