# Supplementary material for: A Qualitative Study to Examine Feasibility and Design of an Online Social Networking Intervention to Increase Physical Activity in Teenage Girls
Source: PLoS One. 2016 Mar 2;11(3):e0150817. doi: 10.1371/journal.pone.0150817 (PMC4774869; doi:10.1371/journal.pone.0150817)
Supplement: S1 Table — (PDF) [file pone.0150817.s002.pdf]

Feasibility and design of an online social networking intervention to increase physical activity in teenage girls

Teenage girls described physical activity as:

**Physical activity is**	**Focus group**	**Physical activity is not**	**Focus group**
Team sports	1, 2, 3, 5, 6	Not walking	1, 3, 4
Running	2, 3, 4, 5	Prefers solo sports	1, 2
Dance	3, 4	Not housework	3
Training program	1, 5	Dislikes running	2
Walking (to friends/transport to school/dog	2, 5	Not dance	6
Lot of fitness and sport	1		
Individual sport	2		
Gym	2		
Routines	2		
Playing with pets	5		
Swimming	5		
Taekwondo	5		
Playing running games such as “red rover”	6		

Product Category defined as the package of benefits that arise from undertaking physical activity

Price Category defined as the cost (barriers) perceived in undertaking physical activity.

**Benefits**	**Focus group**	**Costs**	**Focus group**
**Physical/practical**			
Fitness	1, 3	Time management	1, 2, 4
Health	2, 5	Limited by injury	1
Benefit afterwards	1	School work	2, 4
Motivated to be attractive	2	Paid work	2, 4
		Lack of transport	2
		Lack of money	2
		Weather	2
		Safety (at night)	2
		Other sport commitments	4
		No classes in holidays	4
**Psychological**			

Enjoyment	1, 2, 3, 6	Feel bad about self, due to comparison of performance, not keeping up, burden to team, not good enough	1, 2, 4, 5
Competition	1, 3, 6	Failing to achieve goals on the amount of activity	1
Motivated by others/friends	1, 2, 3, 4	Perceived lack of control (due to training programme?)	1
Self-motivation to achieve personal best	1, 2, 3	Negative messages about weight	2
Do activity with friends	1, 3, 4	Rejection of invitations	5
Social support/talking to a friend/parent while walking	2, 3, 4	Required to motivate friends to join	4
Meet people	2, 4	Responsible for other's motivation	4
Improves mood / happiness	3, 4	Managing a group perceived to be a lot of work	4
Activity supported by parent	1		
Self efficacy – sense of control	1		
Makes you feel better about yourself	5		

Social media as a promotion channel category defined as exploring the effectiveness of various social media formats may be effective for communicating a message about physical activity

**Facilitators**	**Focus group**	**Barriers**	**Focus group**
**Parent perceptions**			
Parent supportive of app	1, 2, 3, 4, 5, 6	Parental concern re social pressure/over use/strangers online	3, 5, 6
Parent will like to be informed	1, 3, 4	Parents won't need to be informed	2
Perceived neutral attitude of parents to online	1, 4	Protective parents	1
Parents will not perceived risk	1	Social media addiction	1
		Social media-rejection	1
		Being forced to take up app	1
**Media platform**			
Facebook	1, 2, 3, 4, 5	None or infrequent Facebook use	6
Instagram	3, 6		

Phone	4, 6		
Computer/ Laptop	2, 4		
ipad	4, 5		

Placement of product category defined as how PA messages are placed or distributed to create opportunities

Facilitators	Focus group	Barriers	Focus group
Promotion through school	1, 4, 5, 6	School not cool	5
Credibility of the university	1	School associated with being boring	4
		School may be judged by non-friends	4
		School opportunity for bullying if fall behind	4

Features of a social media based intervention category defined as factors that might facilitate an increase in physical activity

Facilitators	Focus group	Barriers	Focus group
**Competition**			
Competition element	1, 2, 4, 5, 6	May be too competitive	2
		Pressure to perform	2
**Group setting**			
Do with friends (provide support,) encourage competition	1, 2, 3, 5, 6	Don’t want to be a leader	3, 4, 6
Group consensus process	1, 2, 4, 5	Needing to be friends with strangers	4, 5
Family team	1	Difficulty motivating friends/Friends drop out	4
Sharing roles	5	Falling behind	3
Encourage others to be more active	6	Forgetful	3
Potential to make new friends	6	Friends too kind to motivate effectively	1
Able to communicate with others about progress	2	Group size	2
Safe as only within group	2	Would need others to set up group	6
**Goal setting**			
Goals to get rewards building up points to earn prizes	1, 2, 3, 4	Don’t need reward	1
Reward for joining, sustained effort, reaching levels	2	Unachievable goal	5
Feedback			

feedback on progress, measurement of intensity, periods of activity, time, calorie use, distance	1, 2, 5, 6	Difficult to maintain interest	2
Personalised data	1	Entering information	1
Notification on who is working out at the same time, goals, achievements	2	Errors in measurement device	1
Online reviews from other users	2		
**Features of the programme**			
Variety	2	Technical problems/lack of internet	2, 6
Short duration of programme	2	Daily	3
Timing to ensure relevant to other things that are happening	2	Needing to log in	2
Ability to continue after 50 days	2	Novelty wears off	2
Able to do custom settings re number of notifications and timing of reminders	2	Over exposure	3
Control over choosing to exercise	2		
Incorporates home based exercise	2		
Progression from walking to other exercises	2		
Simple exercises	2		
Weekly challenges	5		
Music	5		

Design category defined as features created by a graphic designer for an online social media program

Facilitators	Focus group	Barriers	Focus group
**Style**			
Colour/bright/light	1, 2, 3, 4, 6	Bland appearance	4, 5, 6
Photos	2, 4, 6	Cartoon	3, 4
“Cool”	1, 3, 5	Too dark	3, 4
Readability/clear font/easy to see	1, 2, 4	Boring design	5, 6
Simplicity	1, 4	Negative association (Ghetto appearance)	1
Fit looking girls	2	Unprofessional look	2
Recognisable title	2	Scrap book look	2
Plain background	3	Looking like a model	2
Eye catching	5	Bright colour	3
		“in your face”	3
		Dull appearance	5
		Plain	5

**Images should**			
Portray a health body image	1, 4, 6	Not be childlike	1, 3, 5
Portray a realistic and appropriate body image	1, 3, 4	Not be too girly	1, 6
Portray a fit image	1, 6	Have no relevance of image to exercise	1, 6
Be age appropriate	1		
Be trustworthy	1		
**Text**			
Relevant tip	1, 2, 3, 4, 5	Boring tip	1, 2, 3, 4
Facts	2, 3, 4, 5, 6	Lame	1
Simple	2, 5	Not relevant to teenage girls	1, 2
Shopping tips	3, 4, 5	Humour	2, 6
Achievable	1,2	Feels forced	1
Motivating tip	1, 6	Mixed messages	1
Useful/effective tip	1	No relevance to exercise	1
Nutrition information	1, 2	Not coherent with other messages	1
Advice on stress management	2	Contradiction between food and exercise	2
Inspirational people	3	Not logical	2
Message about fitness	3	Others story	2
Variety	1	Sounds too old	2
Educational tip	1	Chores tips	3
Tips from role models	1	Inspirational tips that try too hard	3
Inspirational text	2	Shopping tips	6
Short	2		
Addition of other exercise suggestions	2		
Advice on tiredness	2		
Encouraging realistic progress	4		
Motivational	4		

Note: a feature may appear as both a facilitator and a barrier within one focus group reflecting opposing views of girls within the group.
