# Supplementary material for: A Qualitative Study to Examine Feasibility and Design of an Online Social Networking Intervention to Increase Physical Activity in Teenage Girls
Source: PLoS One. 2016 Mar 2;11(3):e0150817. doi: 10.1371/journal.pone.0150817 (PMC4774869; doi:10.1371/journal.pone.0150817)
Supplement: S1 Text — (PDF) [file pone.0150817.s003.pdf]

Feasibility and design of an online social networking intervention to increase physical activity in teenage girls

Focus Group Schedule

Section one

How would you describe your level of enjoyment of physical activity?

Are you are doing as much activity as you want to be doing, or you think you should be doing?

Do you feel you can control how much activity you are doing?

What motivates you to do physical activity?

Section two

Which forms of social media do you use?

How often do you use it?

What sort of devices do you use for access?

<u>(Demonstrate app)</u>

Would you be interested in using an app like this?

    What would entice you?

    What would put you off?

What are your thoughts about this app being associated with school vs out of school?

Would you start a team?

Would you join a team if someone invited you?

What would cause you to drop out?

What would your parents think about you using this app?

Section three

<u>(Present the graphic designs)</u>

What do you think about these?

What kind of picture do you prefer?

Which types of logos do you prefer?

Which colour combinations do you prefer?

(Present cards with physical activity tips)

Could you look at the tips on these cards and reject the ones that you feel are not relevant

Could you write any other tips on the blank cards?

Could you place all of the tips (but not the excluded ones) in order of relevance?

(Present example emails)

Which one do you think is best?
