# Supplementary material for: A Qualitative Study to Examine Feasibility and Design of an Online Social Networking Intervention to Increase Physical Activity in Teenage Girls
Source: PLoS One. 2016 Mar 2;11(3):e0150817. doi: 10.1371/journal.pone.0150817 (PMC4774869; doi:10.1371/journal.pone.0150817)
Supplement: S2 Text — (PDF) [file pone.0150817.s004.pdf]

Enthusiastic

This week, you've logged (total weekly steps) steps. That works out to an average of (average steps) steps for every day you've logged this week.

Great job!!

Do you feel like you can step up to an even bigger challenge?? Why not try increasing your daily step average by an extra 2,000 steps a day? There are lots of awards that you can unlock for step counts above 10,000 a day, PLUS you'll be boosting your team's tally.

OR

Great job! That's a fantastic daily average, just don't forget to keep logging your daily steps!

OR

Pretty good! What do you think you could do to crack the golden 10,000 steps a day? Why not get in touch with your team mates and arrange to be active together– it can be much more fun and motivating that way.

Educational

This week, you've logged (total weekly steps) steps. That works out to an average of (average steps) steps for every day you've logged this week.

That many steps a week is equivalent to burning ______ calories or walking ___% of a marathon!

Your body will have been adapting to the increase in physical activity meaning you can step it up a notch. Continually building on your level of physical activity is called progressive overload, it’s a great way to build fitness and health. Try increasing your daily step average by an extra 2,000 steps a day.

OR

A daily average like that means your muscles will be working more efficiently. They will be contracting more powerfully and extracting oxygen out of your blood better. Don’t forget to keep logging your daily steps.

OR

Pretty good, but let’s see if you can get 10,000 steps or more each day. 10,000 steps helps your cardiovascular and respiratory systems work at their very best.

Motivational/empathetic

This week, you've logged (total weekly steps) steps. That works out to an average of (average steps) steps for every day you've logged this week.

Reflect back on your week, are you happy with how you went? I like to set myself some mini goals to work towards each week so that I can track my progress. Maybe you could try that too?

I'm so impressed! You've really been putting in the hard yards so it's time to challenge yourself. You always want to be pushing those boundaries, I think it's time you try to increase of your daily step average by an extra 2,000 a day.

OR

Nice average! I try to remember, consistency is the key though so keep fitting in that daily physical activity and don't forget to always log your daily steps.

OR

That's a good start but I really want you to achieve 10,000 steps a day. I understand it can be pretty daunting but fitting in little bits of activity through the day really adds up.
